# Determination of the degree of grain refinement in irradiated U-Mo fuels

**DOI:** 10.1016/j.heliyon.2018.e00920

**Published:** 2018-12-08

**Authors:** Andrew M. Casella, Douglas E. Burkes, Paul J. MacFarlan, Edgar C. Buck

**Affiliations:** Pacific Northwest National Laboratory, P.O. Box 999, Richland, WA, 99352, USA

**Keywords:** Materials science, Nuclear engineering

## Abstract

A simple, repeatable method for determination of the degree of grain refinement in irradiated Uranium-Molybdenum fuels has been developed. This method involves mechanical potting and polishing of samples along with examination using a scanning electron microscope located outside of a hot cell. The commercially available software package Mathematica was used to determine the degree of grain refinement by way of a built-in iterative active contour method of image segmentation. Baseline methods for degree of grain refinement assessment are suggested for consideration and further development.

## 1 Introduction

The Materials Management and Minimization Reactor Conversion Program continues to pursue the development of a low-enriched Uranium-Molybdenum alloy fuel to replace high-enriched Uranium-Aluminide and Uranium Oxide fuels for use in civilian research and test reactors. The driver behind this effort is to create a fuel with a higher atom density of Uranium so that a smaller fraction of these atoms need to be ^235^U (lower enrichment) to achieve the same neutronic performance within a reactor. Additionally, it is necessary to demonstrate that the ability of the fuel to retain fission products and reject heat to the reactor coolant does not degrade too significantly as a function of irradiation. This endeavor requires a robust program for fuel development and performance characterization. The typical method of performance characterization involves the irradiation of a test fuel plate followed by post-irradiation examination that usually investigates features such as the size and distribution of fission gas bubbles, interaction layer formation, and thermal and physical properties [1, 2, 3, 4]. Certain analyses of the data and images generated from post-irradiation examination can be quite complex. Thus, development of easily-repeatable baseline methods of analysis that can be directly compared by various researchers to calibrate results for relative comparison is of interest [5, 6, 7, 8]. Development of such baseline approaches not only allows for easier comparison of results from various efforts, but also allows for easier communication of more complex analytical methods that can be presented as deviations from baseline methods. Baseline methods have been recently suggested for the analysis of fission gas bubble size, distribution, number, eccentricity, and orientation [8]. These methods utilized intensity threshold methods for image segmentation and identification of fission gas bubbles for subsequent analyses [9]. As will be discussed in this paper, this method of image segmentation is not applicable to efforts to assess degree of grain refinement. Instead, a contour segmentation method is required [10, 11, 12]. This paper presents a suggested baseline method for the assessment of degree of grain refinement of irradiated U-Mo fuel and provides suggestions for future use and enhancement of the method. This method is suggested as an easily repeatable companion method to another recently published method [13].

**Fig. 1:**
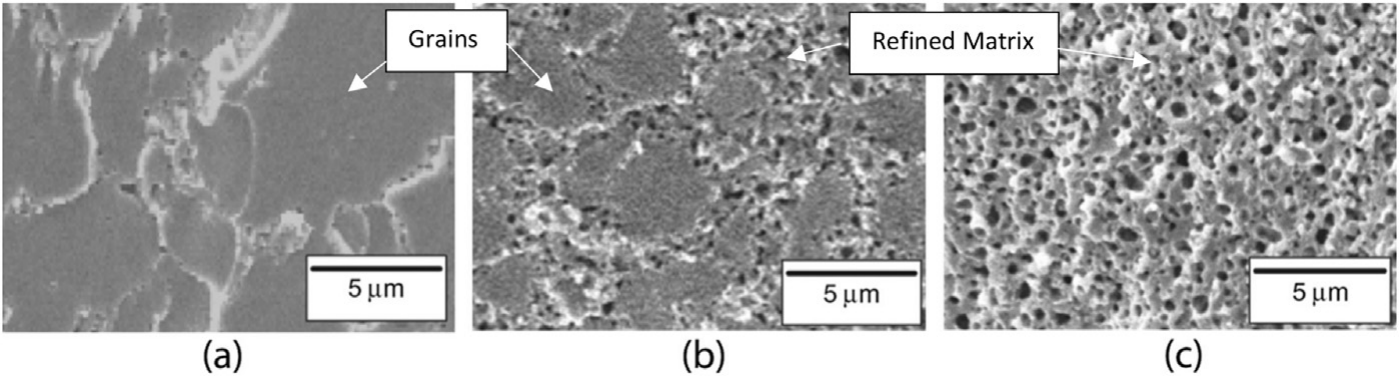
SEM micrographs showing the gas bubble evolution with burnup in U-10 wt% Mo showing fuels with (a) fission density of 2.4 × 10^27^ f/m^3^; (b) fission density of 2.9 × 10^27^ f/m^3^; (c) fission density of 5.5 × 10^27^ f/m^3^. At lower fission densities, the microstructure is composed of mostly intact grains with fission gas bubbles populating the grain boundaries. As the fission density increases, the fuel undergoes “grain refinement” in which a growing portion of the microstructure is comprised of large fission gas bubbles and U-Mo that is no longer part of observable grains (refined matrix). Additionally, the size of the remaining intact grains decreases. ^∗^Reprinted from J. Nucl. Mater., 462, S. Hu, A.M. Casella, C.A. Lavender, D.J. Senor, and D.E. Burkes, Assessment of effective thermal conductivity in U-Mo metallic fuels with distributed gas bubbles, 64–76, 2015, with permission from Elsevier ^∗∗^Reprinted from J. Nucl. Mater., 419, Y.S. Kim and G.L. Hofman, Fission product induced swelling of U-Mo alloy fuel, 291–301, 2011, with permission from Elsevier.

The first step in developing a suggested baseline method for assessment of degree of grain refinement is to define the degree of grain refinement. Fig. 1 presents the structures relevant to this discussion [14, 15], with grain refinement being the reduction in the size of grains coinciding with the formation of refined matrix. It has long been realized that the behavior of fission gas bubbles as they form and migrate within fuels during irradiation has a significant impact on fuel performance. In particular, as the degree of grain refinement increases, the thermal conductivity of the fuel decreases. Fission gas behavior has been characterized as a function of fuel matrix, burnup, and irradiation temperature. For γ-phase Uranium alloys such as U-Mo that have been irradiated to high burnup at low irradiation temperatures, the bulk effect of fission gas generation is categorized as “grain refinement with bubble growth on the newly formed grain boundaries” [16]. This phenomena occurs for U-Mo fuels irradiated at temperatures below 250 °C at a burnup of around 3 × 10^21^ fissions/cm^3^ [13]. Observation of U-Mo fuels that have been subjected to this irradiation regime appear to consist of intact grains floating within a sea of refined matrix that contains a high density of fission gas bubbles. For an image taken of a cross section of such a fuel, the degree of grain refinement is defined as the ratio of the image area composed of the gas bubble-containing refined matrix to the total area of the image. The difficulty in determining this ratio lies in accurately identifying intact grain boundaries and the area contained within the shapes they enclose. Manual identification of these intact grain boundaries is an exhaustive procedure that is subject to subjective criticism. An automated method allows for a foundational assessment from which more objective critiques can be formed based on the quantitative values of parameters associated with the method; allowing for uniform baseline assessments from the many researchers across this field. This paper proposes one such baseline method.

## 2 Material and methods

The materials and methods of preparation utilized in this paper have been thoroughly discussed in a recent publication [8]. A summary of this discussion is repeated here for convenience of the reader. The sample that is studied in this paper originated from an Advanced Test Reactor (ATR) Full-size Plate in Center Flux Trap Position-3 (AFIP-3) which consisted of AA6061 clad monolithic fuel plates using a metallic foil of U-10Mo enriched to nominally 19.75% ^235^U. The metallic fuel foil of interest in this study was prepared by hot co-rolling a U-10Mo alloy ingot with Zr on either side [17]. The foil was hermetically sealed and bound within the AA6061 cladding using a hot isostatic pressing (HIP) process [18] and irradiated at the ATR located at Idaho National Laboratory (INL). Additional details on the various aspects of the AFIP-3 experiment can be found in Perez et al. [19].

Post-irradiation, rectangular segments (roughly 12.5 mm × 27 mm) were taken from the AFIP-3BZ (referred to as Segment E) fuel plate at INL and transferred to Pacific Northwest National Laboratory (PNNL) for examination of thermal properties [20]. The analyses performed at PNNL provided information regarding thermal transport and fission gas release on the physical scale of several millimeters [1, 21]. Additionally, these analyses provided data and insight for efforts to model microstructural evolution during irradiation and the subsequent impact on thermal properties [14]. However, more detailed information regarding microstructure and fission gas bubble distribution is necessary than was possible to acquire with an optical microscope. To obtain this detailed information, potted metallography samples were resurfaced and examined with scanning electron microscopy (SEM).

Fuel segment E (sample TE), taken from the fuel plate irradiated in experiment AFIP-3BZ, was received at PNNL in February 2014. The AFIP-3BZ fuel plate had in initial ^235^U enrichment of 19.937%, an initial Mo concentration of 10.3 wt%, and was irradiated in the ATR for 101.0 Effective Full Power Days (EFPD) during which it reached an average burnup of 63.5 at% and a calculated fission density of 4.32 × 10^21^ fissions per cm^3^ [1,20]. Segment E was sectioned into smaller pieces at PNNL and each piece was designated for a particular measurement within the suite of examinations necessary for robust thermal analysis (optical metallography, laser flash analysis, gas pycnometry, and differential scanning calorimetry). TE-OM1 was a piece that was mounted in epoxy, examined with optical microscopy, and placed in storage. During storage, the epoxy experienced radiation damage and the sample had to be re-surfaced. The same method was used for the initial preparation and re-surfacing of TE-OM1 and is documented elsewhere [22].

## 3 Theory/calculation

**Fig. 2:**
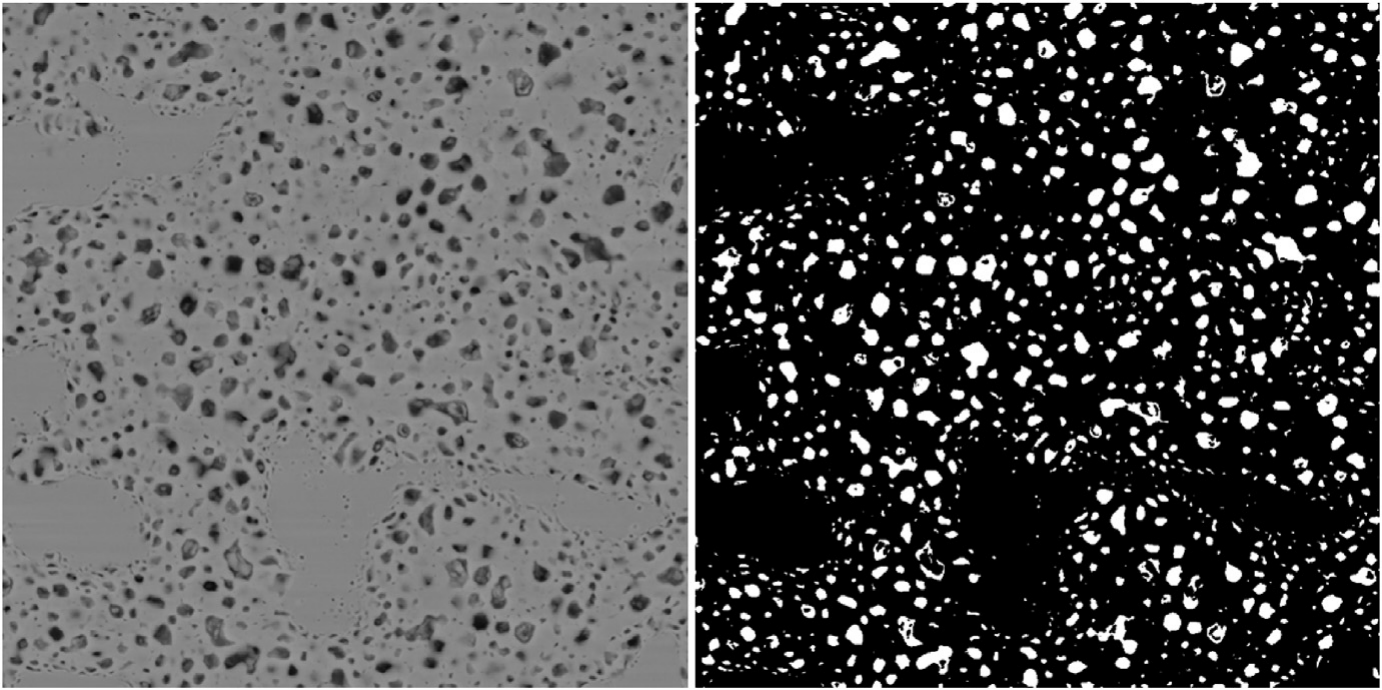
Comparison of SEM image (left) as captured by the instrument with the image generated with an Otsu segmentation (right).

Recently, the authors published a method for a simple, rapid, repeatable characterization of fission gas bubbles in the same SEM images considered in this paper [8]. That method applied the Otsu approach for image segmentation by way of an intensity threshold. However, this same method was found to be ineffective in the characterization of grains themselves. The reason for this ineffectiveness is apparent upon comparison of an SEM image to the segmented image generated from it by the method under current discussion. An example of two such images is given in Fig. 2 [23]. As can be seen, the method clearly separates the fission gas bubbles from the refined matrix and remaining grains, allowing for object identification. However, the refined matrix is indistinguishable from the remaining grains as everything that is not dark enough to be a fission gas bubble in the original image is rendered black in the segmented image. Thus, an attempt to segment the image results in one large grain with a multitude of internal bubbles. It is possible that an appropriate threshold segmentation scheme could be developed to isolate the grains themselves, but the authors could not find any scheme that met the objective of the effort to determine a simple, rapid, repeatable method for doing so. A survey of the literature revealed that other researchers rely on direct measurement methods and a lineal intercept method [13, 24]. In the direct method, the size of each grain is determined by measuring it from four directions and then averaging the results. In the lineal intercept method, several straight lines are drawn in different directions on a fuel particle cross section and grain size is determined from line length and intersections with grain boundaries [24, 25]. These methods are time consuming and can produce problems with repeatability.

It is clear that to avoid methods such as direct measurement and the lineal intercept method, it is necessary to develop a method for isolating the remaining grains in much the same way as the Otsu segmentation isolated fission gas bubbles. To do this, a contour segmentation method was employed in place of an intensity threshold method. In keeping with the same methodology as our previous study focused on characterizing fission gas bubbles, the authors searched existing methods within the commercially available software packages CellProfiler, MATLAB, and Mathematica [26, 27, 28]. One such method that was found was the Chan-Vese segmentation algorithm for processing noisy images [10, 11, 12]. Mathematica contains the built-in function “ChanVeseBinarize” that segments an image by computing optimal contours around regions of consistent intensity in the image [28]. Activecountour(A, mask) is available in MATLAB for segmentation according to Chan-Vese or “edge” segmentation, but the authors did not investigate this option for comparison and contrast with the option available in Mathematica.

Per Mathematica documentation [28], “The Chan-Vese segmentation of an image domain Ω into the two segments D and Ω∖D with contour Γ = ∂D minimizes the following functional F of image *f*:”

(1) $$F\left(c_{1},c_{2},\text{Γ}\right)=\text{μ}\;\text{Length}\left[\text{Γ}\right]+\;\nu \;Area\left(D\right)+\lambda_{1}{\int^{}}_{D}{\left|f-c_{1}\right|}^{2}dxdy+\lambda_{2}{\int^{}}_{\Omega \setminus D}{\left|f-c_{2}\right|}^{2}dxdy$$

The minimization of F is an iterative process and the resulting segmentation of *f* is influenced by the values of the weighting coefficients μ, ν, λ_1_, and λ_2_ identified as the length penalty, area penalty, and level penalties respectively. The effects of optimal and non-optimal choices of these coefficients, as well as the number of iterations performed in a given minimization operation has been addressed in the literature [12]. The Mathematica default values for these parameters are μ = 0, ν = 0.03, λ_1_ = 1.0, and λ_2_ = 1.0.

Following a similar procedure to that used for image processing to characterize fission gas bubbles [8], the sequence of Mathematica commands used to process an image for degree of refinement determination is:

I1=Import[“path∖∖I0”];
I2=ImageCrop[I1,{850,850},{Right,Bottom}];
I3=ChanVeseBinarize[I2,"LevelPenalty"→{x,1},MaxIterations→y]

As can be seen by the third command, the default value for λ_1_ had to be adjusted from 1 and the default number of iterations that the segmentation algorithm is executed is denoted as a variable that has to be adjusted from the default value of 100 in order to ensure convergence. Many other parameter adjustments can be applied to produce equivalent or arguably better segmented images. Additionally, application of an image filter such as a Gaussian filter prior to execution of the Chan-Vese segmentation was also observed to generate images with objects containing softer edges. However, in keeping with the stated goal of the work presented in this paper, the three-command sequence presented above with two default parameter adjustments is the simplest acceptable process the authors could devise. Once a satisfactory segmentation of the image has been accomplished, the degree of refinement can be calculated by determining the fraction of pixels in the image assigned to foreground (surviving grains) and the fraction that is assigned to background (refined matrix).

**Fig. 3:**
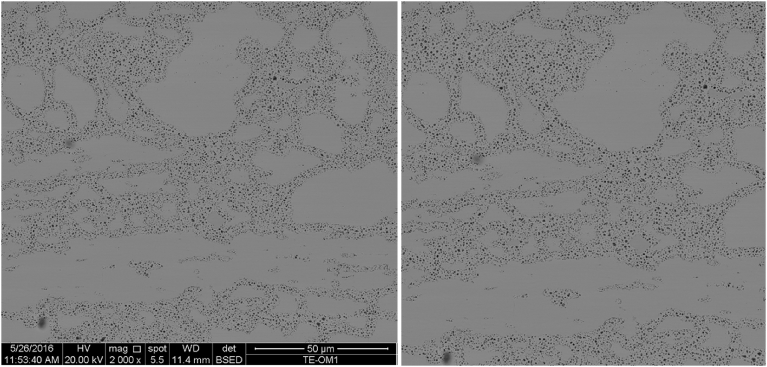
Image taken from position 3 at 2000x magnification with associated information and scale bar (left) and cropped for analysis (right).

In the analyses presented in this paper, SEM images of magnifications of 2000x, 5000x, 10000x, 15000x, 20000x, and 25000x were acquired at five separate locations across the center line of the fuel meat. Fig. 3 shows the image acquired at 2000x at position 3 before and after the ImageCrop step mentioned above. This step is necessary to remove the image information bar included at the bottom of the acquired image. There is benefit to viewing the two images shown in Fig. 3 next to one another as one provides a scale bar and includes all features captured in the image, while the cropped image shows the exact image that is being segmented and analyzed.

**Fig. 4:**
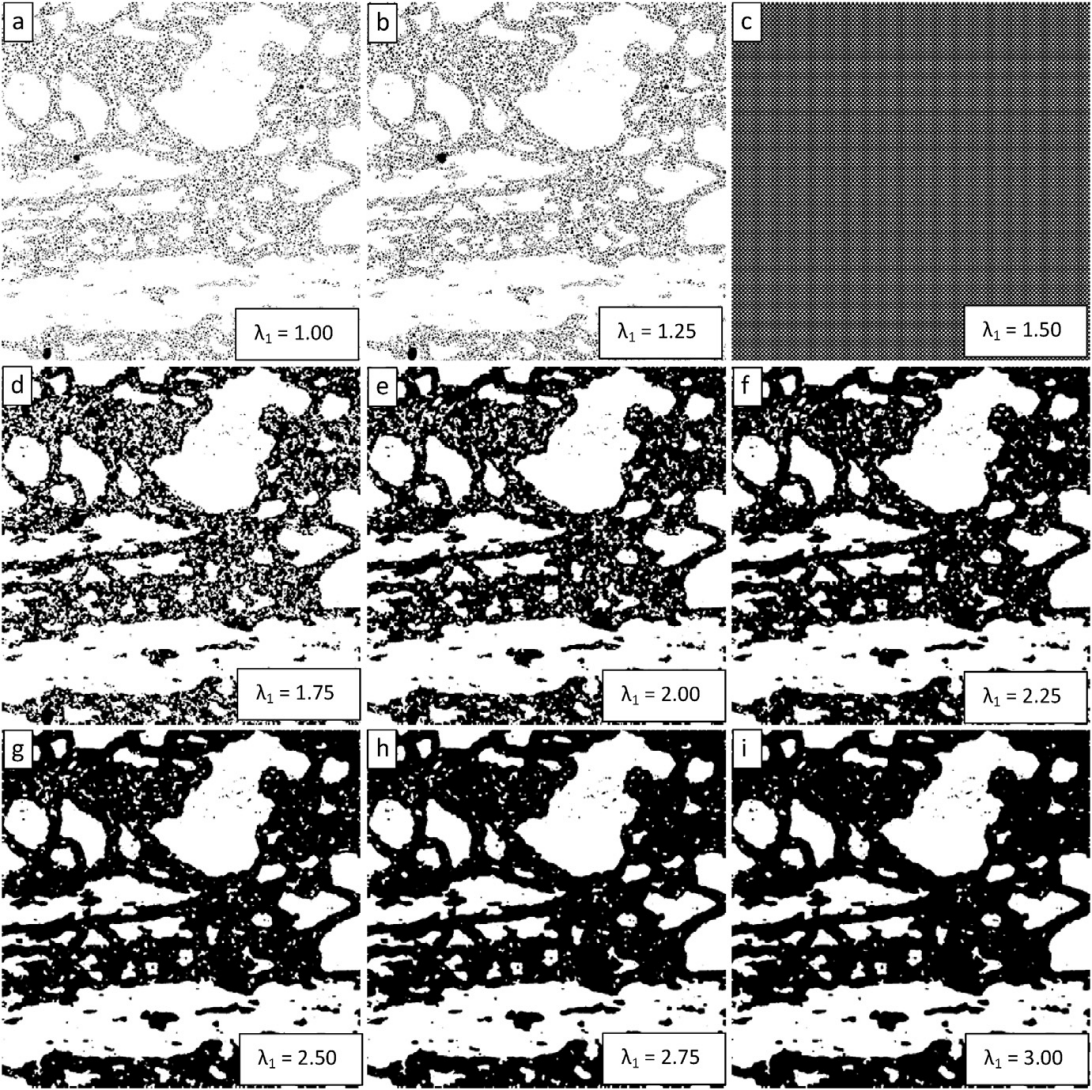
Demonstration of the convergence of the Chan-Vese algorithm on the image shown in Fig. 3 for μ = 0, ν = 0.03, λ_2_ = 1.0, MaxIterations = 150, and λ_1_ varying from 1 to 3 in steps of 0.25 from upper left to lower right (a to i).

The image on the right-hand side of Fig. 3 was segmented several times using the ChanVeseBinarize command with MaxIterations set equal to 150 and the value of λ_1_ varying from 1 to 3 by steps of 0.25. The results are presented sequentially from the top left image to the bottom right image in Fig. 4. Fig. 4 demonstrates that there is a threshold value of λ_1_ below which the algorithm does not achieve a successful segmentation. This threshold appears to be in the vicinity of 1.50, where the algorithm fails to generate segmented images. For λ_1_ values below this value, the segmentation is poor. For λ_1_ values above this value, the quality of the segmentation (as defined as a visual match to the desired segmentation when viewing the right-hand image of Fig. 3) appears to increase as λ_1_ increases.

**Fig. 5:**
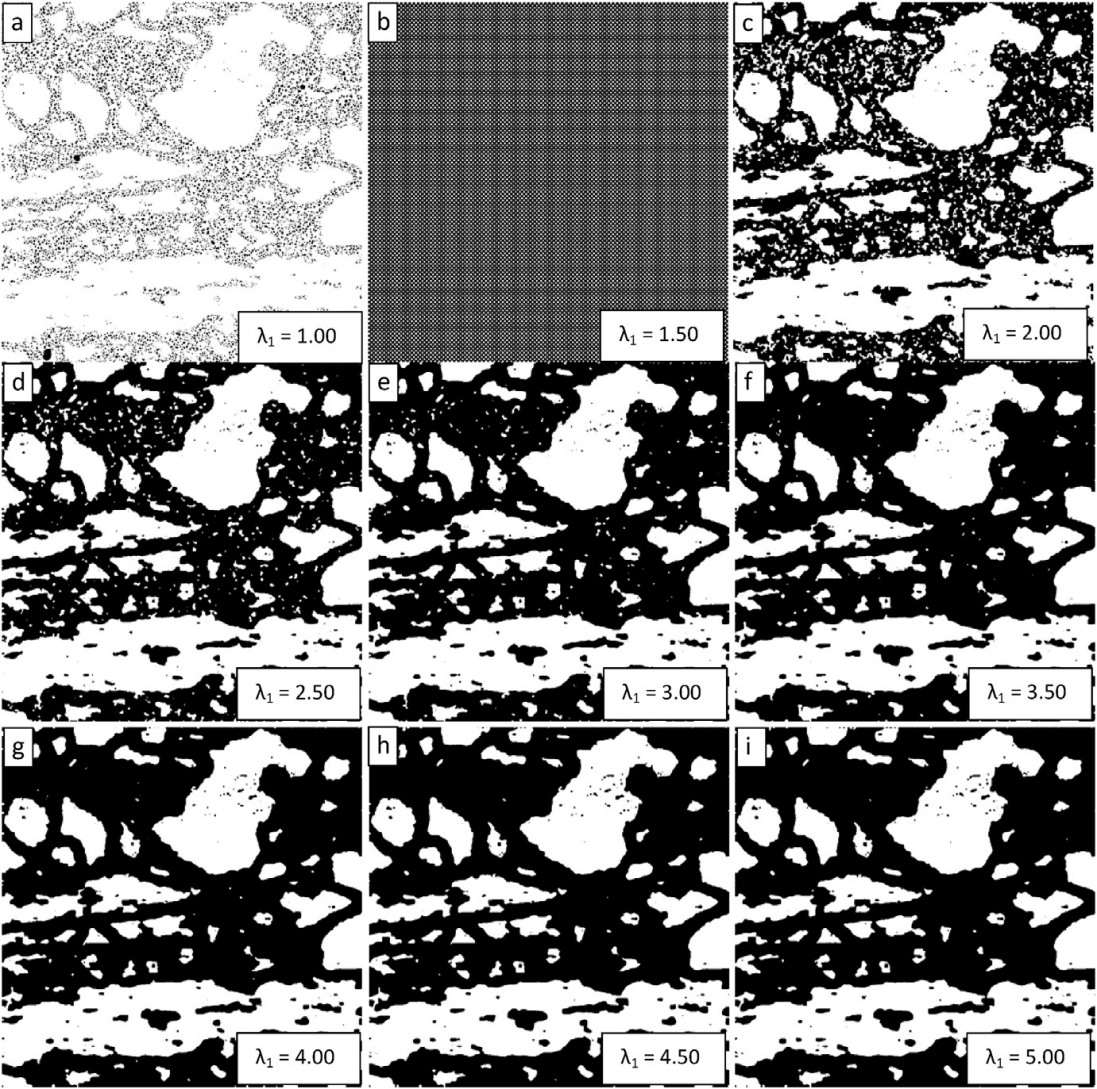
Demonstration of the convergence of the Chan-Vese algorithm on the image shown in Fig. 3 for μ = 0, ν = 0.03, λ_2_ = 1.0, MaxIterations = 150, and λ_1_ varying from 1 to 5 in steps of 0.5 from upper left to lower right (a to i).

The parametric study of the resulting image segmentations on varying the value of λ_1_ was expanded from that in Fig. 4 to a range from 1 to 5 in steps of 0.5 in Fig. 5. Fig. 5 shows the same threshold value of λ_1_ = 1.50 and shows that the apparent quality of the segmentation continues to improve beyond a value of 3. However, the extent of the improvement appears to decline with the improvement between λ_1_ values of 4.5 and 5 being minimal.

**Fig. 6:**
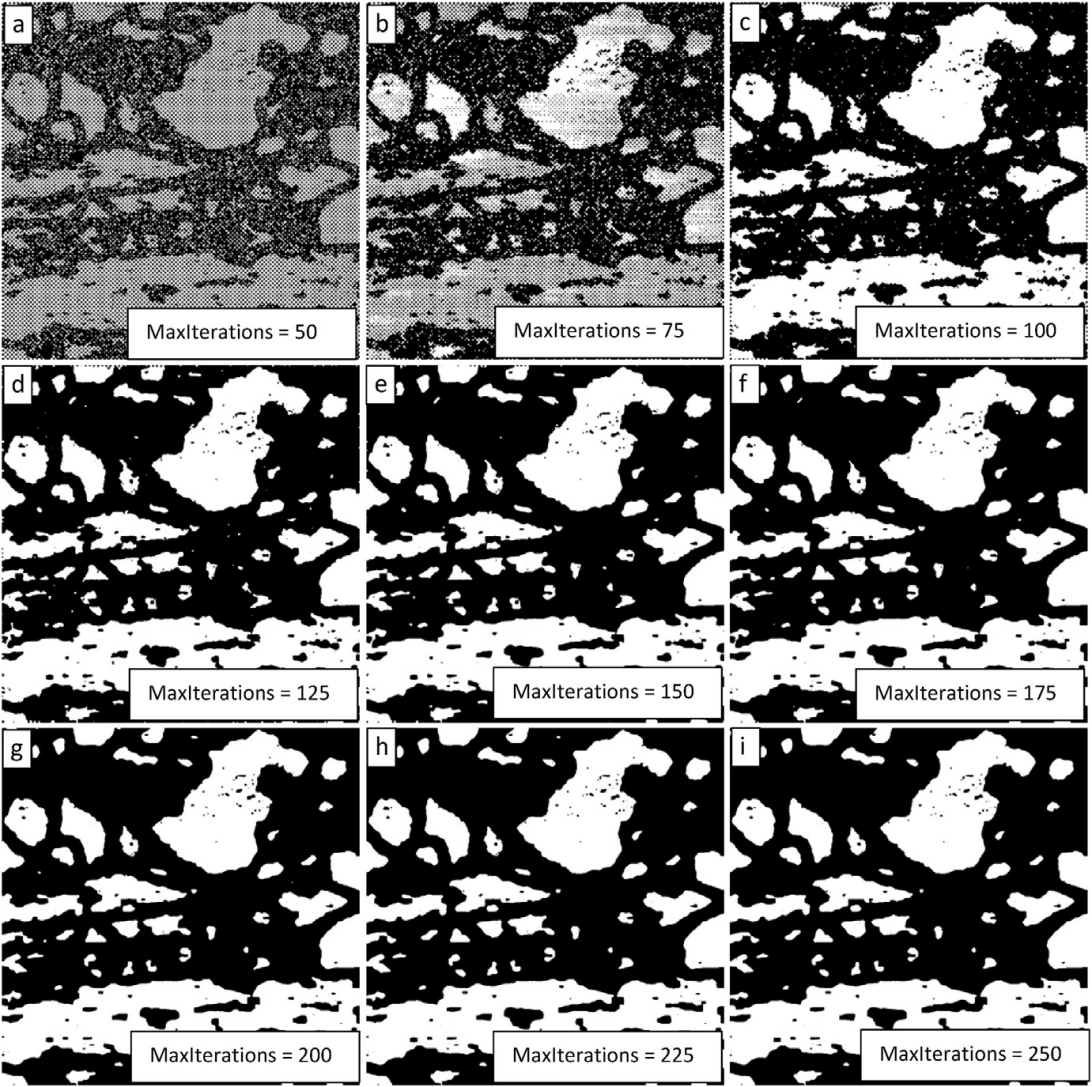
Demonstration of the convergence of the Chan-Vese algorithm on the image shown in Fig. 3 for μ = 0, ν = 0.03, λ_1_ = 5, λ_2_ = 1.0, and MaxIterations varying from 50 to 250 in steps of 25 from upper left to lower right (a to i).

Having determined that a value of λ_1_ = 5 generates a quality segmentation, that value is locked in and a parametric assessment of the impact of the value assigned to MaxIterations is initiated. The results for the value of MaxIterations varying from 50 to 250 in steps of 25 are shown in Fig. 6. Again, there appears to be a threshold between MaxIterations = 75 and 100 above which a successful segmentation can be obtained. The segmentation quality appears to improve with higher values of MaxIterations, but the extent of this improvement is not perceptible above a value of 150.

**Fig. 7:**
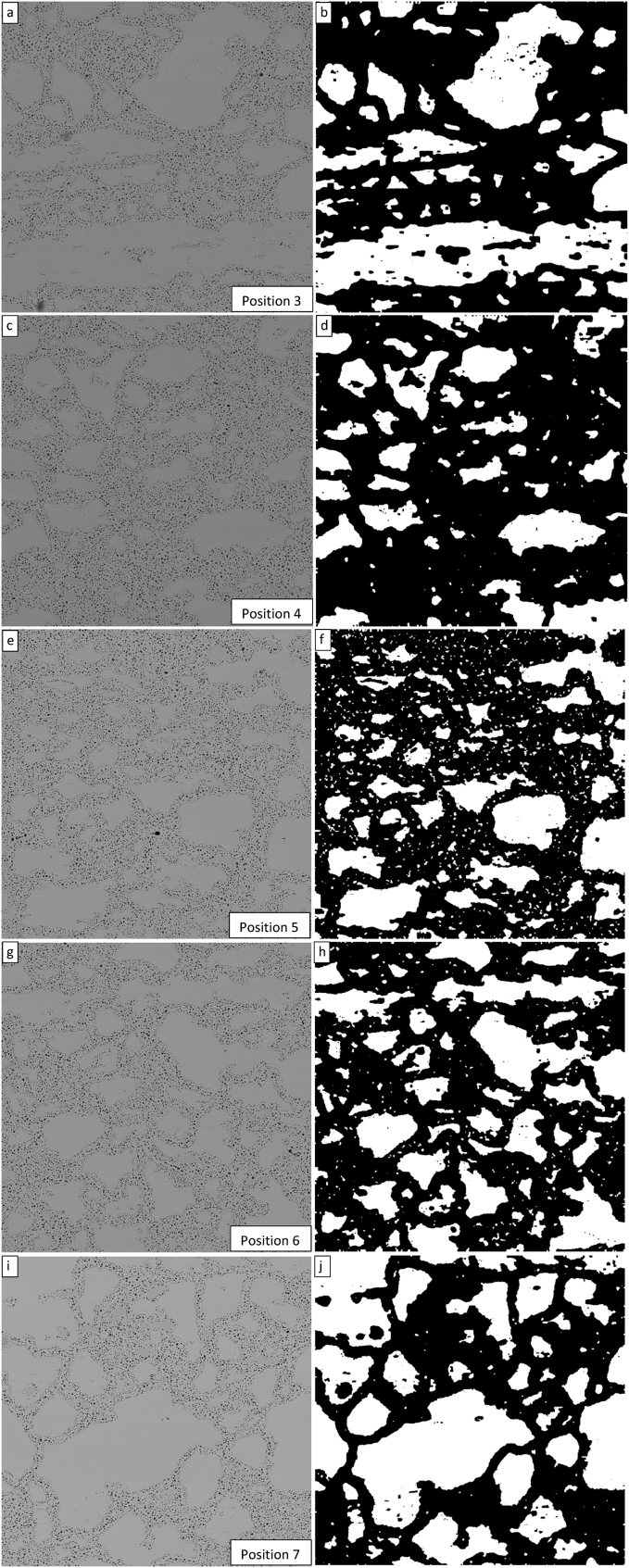
Demonstration of the Chan-Vese contour segmentation for μ = 0, ν = 0.03, λ_1_ = 5, λ_2_ = 1.0, and MaxIterations = 150 for all five fuel locations examined progressing from position 3 (a) to position 7 (i). All original images occurring in this figure were acquired at 2000x magnification.

Having determined that the values of λ_1_ = 5 and MaxIterations = 150 result in a quality segmentation for the 2000x magnification image at position 3 of the sample, these same values were used to segment the 2000x magnification images taken at the other four locations that were examined. In total these 5 locations are identified as positions 3, 4, 5, 6, and 7, with positions 1 and 2 being the edges of the fuel sample that were anchored to determine the equal spacing distances between the five locations examined within the sample. The results of these segmentations are presented in Fig. 7.

The images in Fig. 7 show that the segmentation process was successful for all images. In some cases (the image from position 5 in particular), there appear to be small pieces of remaining grains within the refined matrix. It is challenging to judge whether these small pieces are actually remaining intact grains or if the algorithm has not segmented such images as well as others. The parameters associated with the algorithm can be adjusted as was done in Fig. 3 through Fig. 6 above for λ_1_ and MaxIterations or for μ, ν, and λ_2_. However, in this analysis, the same algorithm was applied to all images for consistency in comparison.

Once an image has been segmented as demonstrated in Fig. 7, the “degree of grain refinement” or fraction of the original grain structure that has been converted to refined matrix and fission gas bubbles can be determined from the number of white and black pixels. As each image (post cropping) consists of a square matrix of 850 × 850 pixels, the degree of grain refinement in a segmented image is determined by

(2) $$Degree\;of\;grain\;refinement=1-\;\frac{Total\;number\;of\;white\;pixels\;in\;the\;segmented\;image}{850^{2}}$$

The results of an image analysis need only present the values of the parameters associated with the algorithm listed above, a comparison of the original and segmented images, and the final value of the degree of grain refinement for a thorough review and comparison to be performed by other researchers in the field.

## 4 Results

Following the method laid out in the previous section, the degree of grain refinement was determined for each of the positions at a magnification of 2000x as reported in Table 1. The average of the values presented in Table 1 is 0.646 and the standard deviation is 0.083. The values at positions 3, 5, and 6 are relatively consistent at this value, while the value at position 4 is considerably higher and the value at position 7 is considerably lower. Visual examination of the images indicates that this is due to the lack of large grains in the image from position 4 and the presence of many large grains in the image from position 7. This indicates that the results are location dependent and there is an image sample size bias leading to the need to average the results of many images taken at this magnification to obtain a result that is characteristic of a sample of interest. This observation also indicates that high resolution images at lower magnifications may be more valuable for this analysis than high magnification images.

**Table 1:** Degree of grain refinement for 2000x magnification images at all positions examined.

Position	3	4	5	6	7
Degree of Grain Refinement	0.625	0.754	0.686	0.637	0.530

**Fig. 8:**
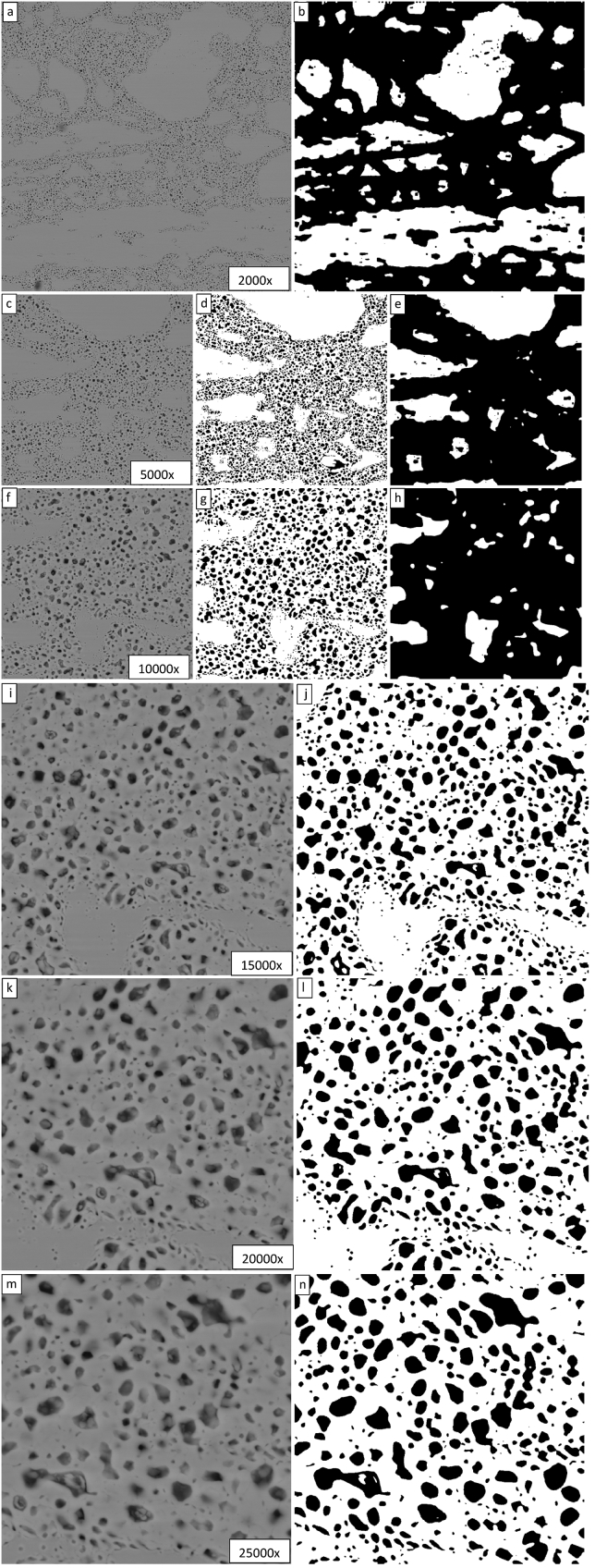
Demonstration of the Chan-Vese contour segmentation for μ = 0, ν = 0.03, λ_1_ = 5, λ_2_ = 1.0, and MaxIterations = 150 for all six magnifications at position 3 progressing through the magnifications (2000x, 5000x, 10000x, 15000x, 20000x, 25000x) from top to bottom (a to m). Further method refinement: 5000x (λ_1_ = 6.5 and MaxIterations = 1000); 10000x (λ_1_ = 3 and MaxIterations = 500, length penalty = 0.25).

When characterizing fission gas bubbles, it was discovered that higher magnification images yielded additional information. As these same images were available for analysis in the current study, the images at position 3 for all magnifications were subjected to the same method of analysis with the results shown in Fig. 8. The same analysis parameters used in the generation of Fig. 7 did not yield successful segmentations for all images. In fact, at 5000x, the next highest magnification available, the level penalty had to be increased from 5 to 6.5 and the MaxIterations value had to be increased to 1000 to achieve a successful segmentation. At 10,000x magnification, a successful segmentation could be achieved for a level penalty of 3 and a MaxIterations value of 500, but the length penalty had to be increased from 0.03 to 0.25. For the magnifications of 15000x, 20000x, and 25000x, the method could not be easily used to generate a successfully segmented image. The degree of refinement for the 2000x, 5000x, and 10000x images shown in Fig. 8 are 0.625, 0.725, and 0.870 respectively. For magnifications above 10000x, the algorithm works to define the contour of bubbles instead of grains as they have become the dominant features in the image.

The images in Fig. 8 demonstrate that to effectively determine the degree of grain refinement of an irradiated U-Mo fuel specimen, an image must be acquired that contains a relatively large field of view. For the SEM image set available for characterization presently discussed, 2000x is the minimum magnification available. While these images can be segmented fairly easily, it is necessary to characterize many of them to get an understanding of the degree of grain refinement to be attributed to the bulk fuel. There is significant deviation in the degree of grain refinement at each of the five locations examined in this study. For future studies, lower magnification images with wider fields of view would be recommended. This recommendation begs the question of whether the use of scanning electron microscopy is necessary for the characterization of degree of grain refinement as it was for the characterization of fission gas bubbles. Perhaps contour segmentation techniques are hindered by the abundance of features not associated with grain boundaries that appear in the SEM images, and OM images may provide a better canvas for segmentation. Images of the fuels examined in this paper had been previously acquired with an optical microscope housed within a hot cell [23]. The contour segmentation technique discussed in the current paper was applied to one of these images to investigate effectiveness on images acquired with OM vs. those acquired with SEM.

**Fig. 9:**
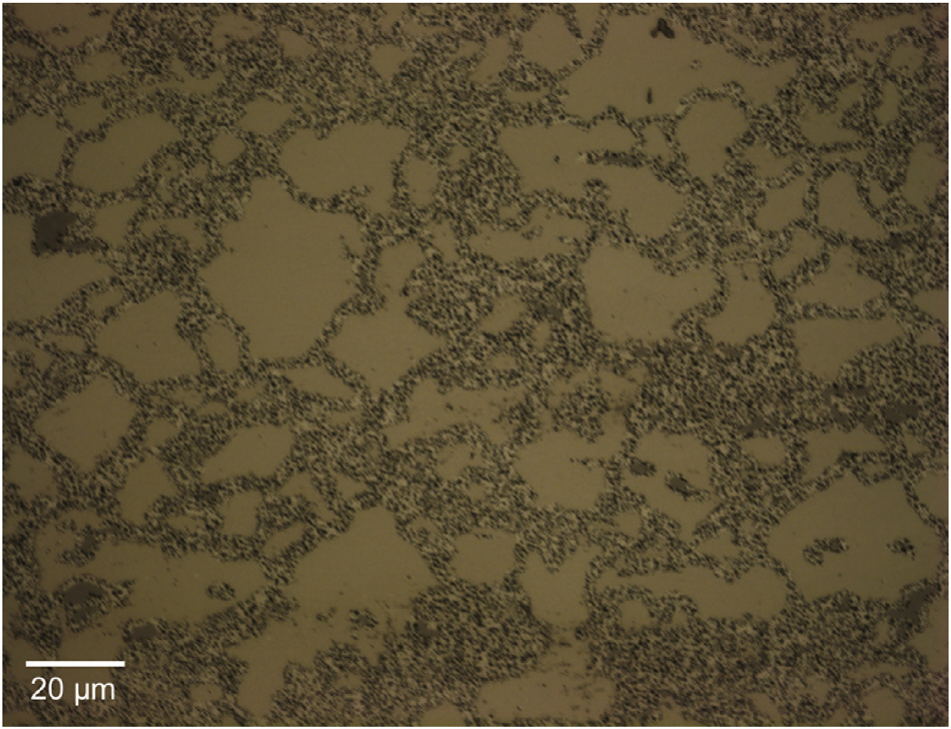
Low magnification image of TE-OM1 captured with an optical microscope housed within a hot cell.

**Fig. 10:**
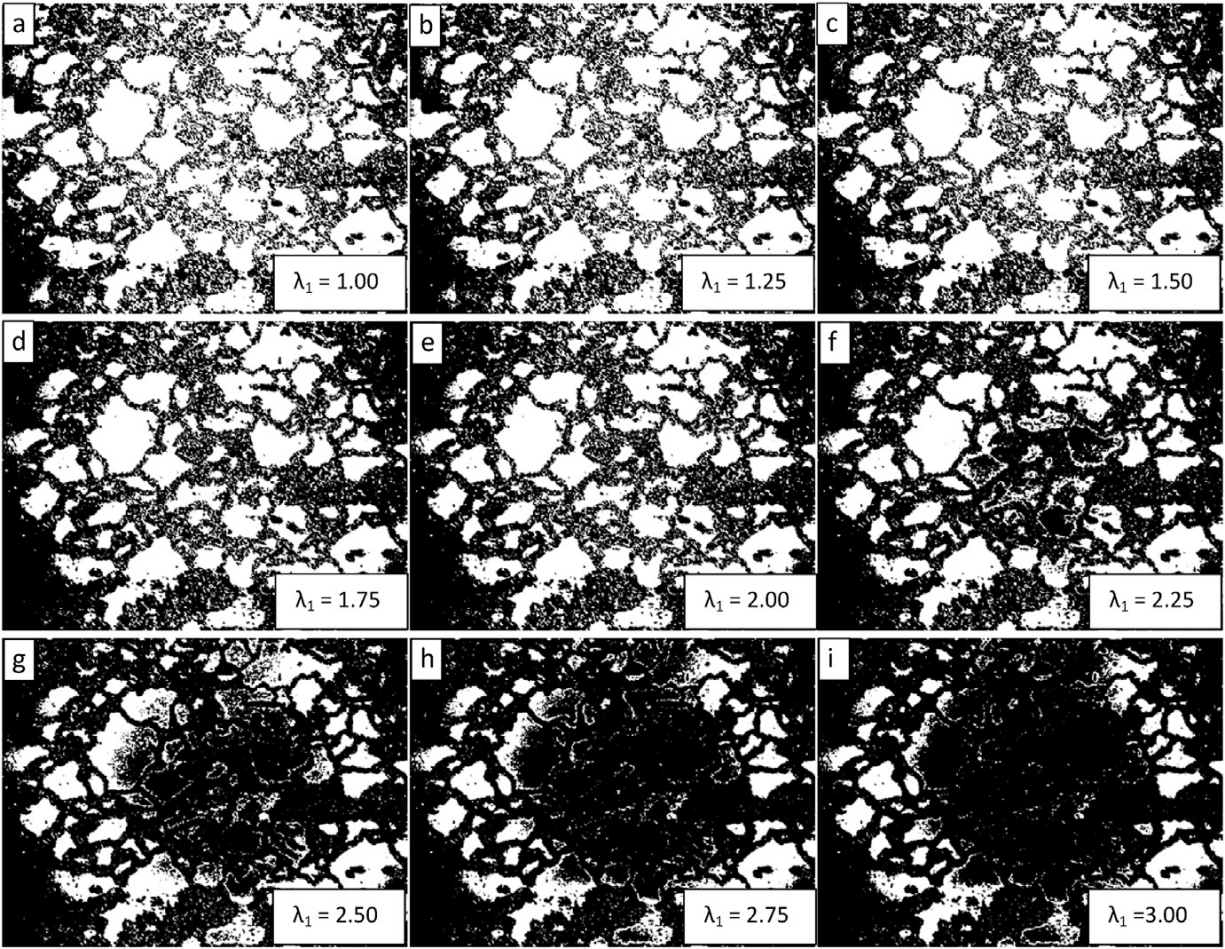
Demonstration of the convergence of the Chan-Vese algorithm on the image shown in 9 for μ = 0, ν = 0.03, λ_2_ = 1.0, MaxIterations = 150, and λ_1_ varying from 1 to 3 in steps of 0.25 from upper left to lower right (a to i).

Fig. 9 shows an optical image of the TE-OM1 fuel. Following the same procedure for investigating the segmentation parametric space presented above, the results of varying the level penalty from 1 to 3 with steps of 0.25 while setting the value for the MaxIterations to 150 are shown in Fig. 10. Of the segmentations presented in Fig. 10, the one corresponding to a level penalty value of 2 appears to be the best, with larger quantities of refined matrix not being removed for lower values and intact grains being overly eroded for larger values. In all cases, intact grains are removed along the image periphery. This is likely an artifact of inhomogeneities in image illumination during acquisition.

**Fig. 11:**
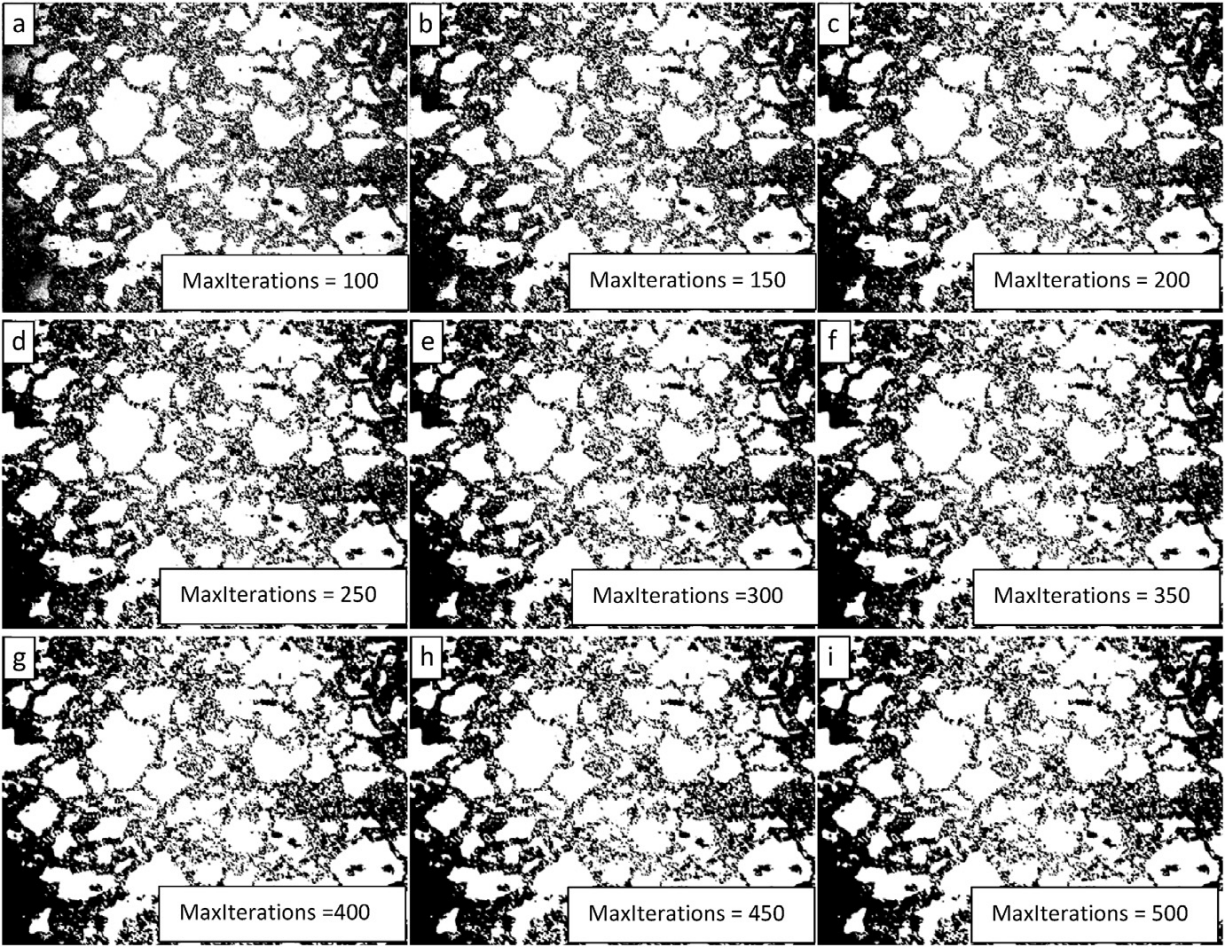
Demonstration of the convergence of the Chan-Vese algorithm on the image shown in 9 for μ = 0, ν = 0.03, λ_1_ = 1, λ_2_ = 1.0, and MaxIterations varying from 100 to 500 in steps of 50 from upper left to lower right (a to i).

**Fig. 12:**
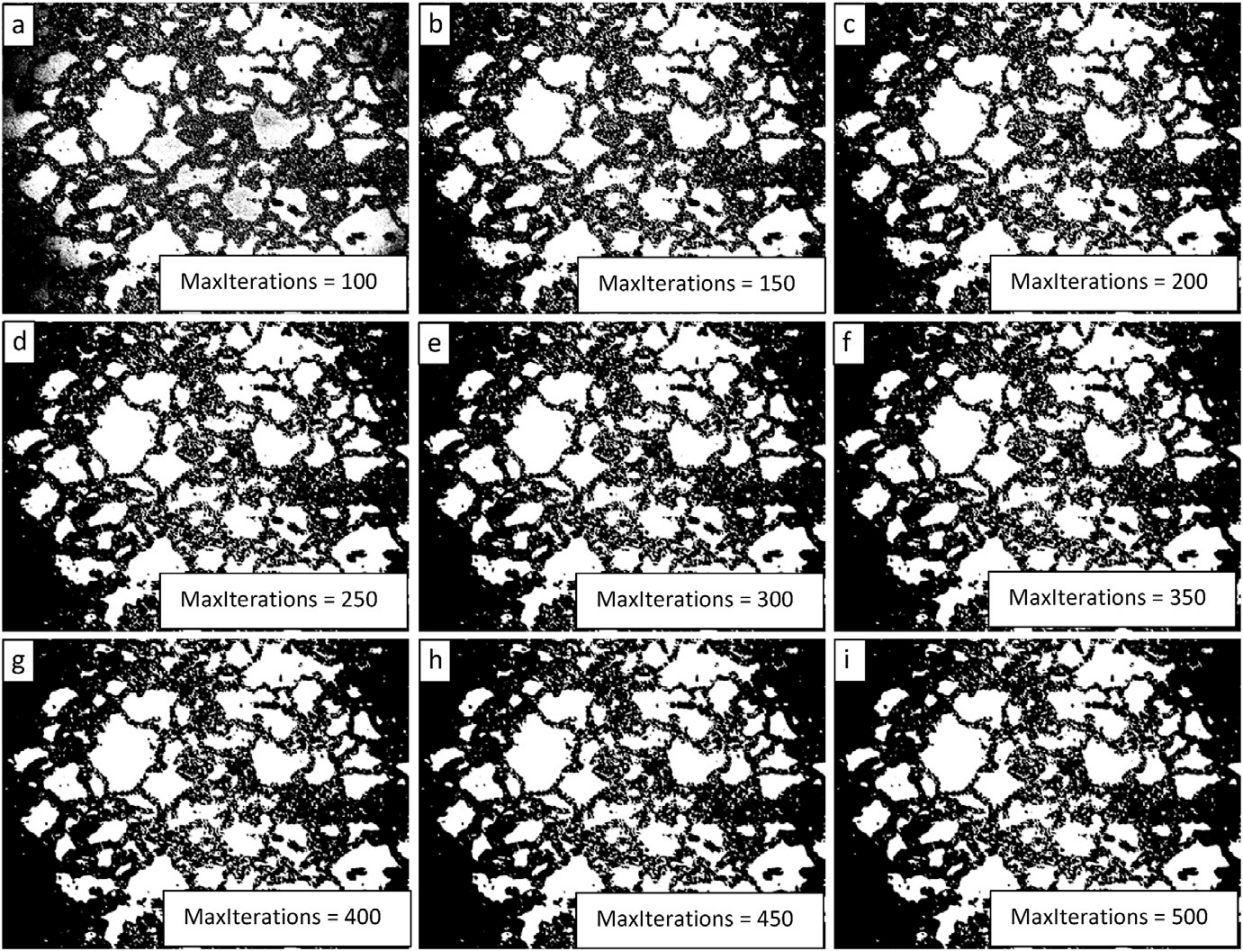
Demonstration of the convergence of the Chan-Vese algorithm on the image shown in Fig. 9 for μ = 0, ν = 0.03, λ_1_ = 2, λ_2_ = 1.0, and MaxIterations varying from 100 to 500 in steps of 50 from upper left to lower right (a to i).

Based on the results of the parametric study of the level penalty, subsequent parametric studies of the MaxIterations with level penalty values of 1 and 2 were completed. The results of these studies are presented in Figs. 11 and 12 and show that a level penalty of 2 provides a better segmentation in the interior of the image and that algorithmic erosion of intact grains continues at the image periphery for a level penalty of 1 or 2 (though it is less pronounced for a level penalty value of 1). There is no apparent benefit for either level penalty value of increasing the value for the MaxIterations beyond a value of 300.

**Fig. 13:**
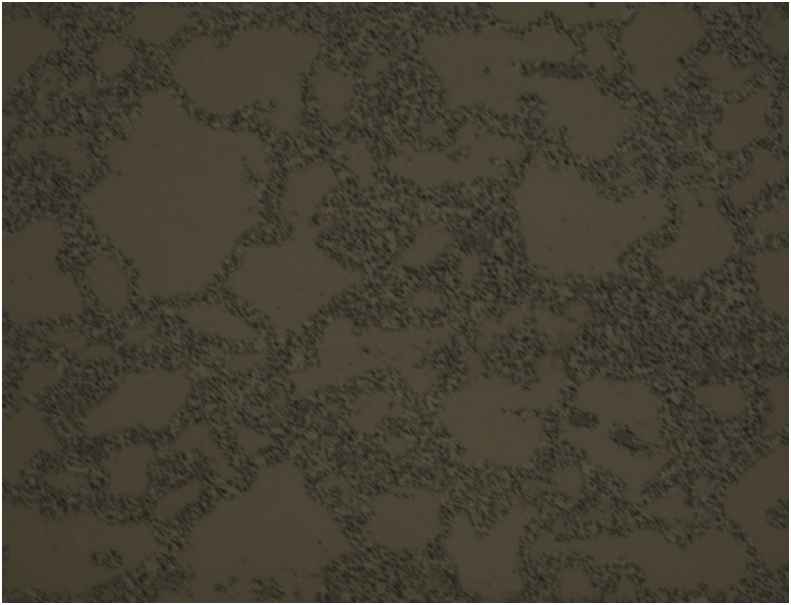
Cropped OM image.

**Fig. 14:**
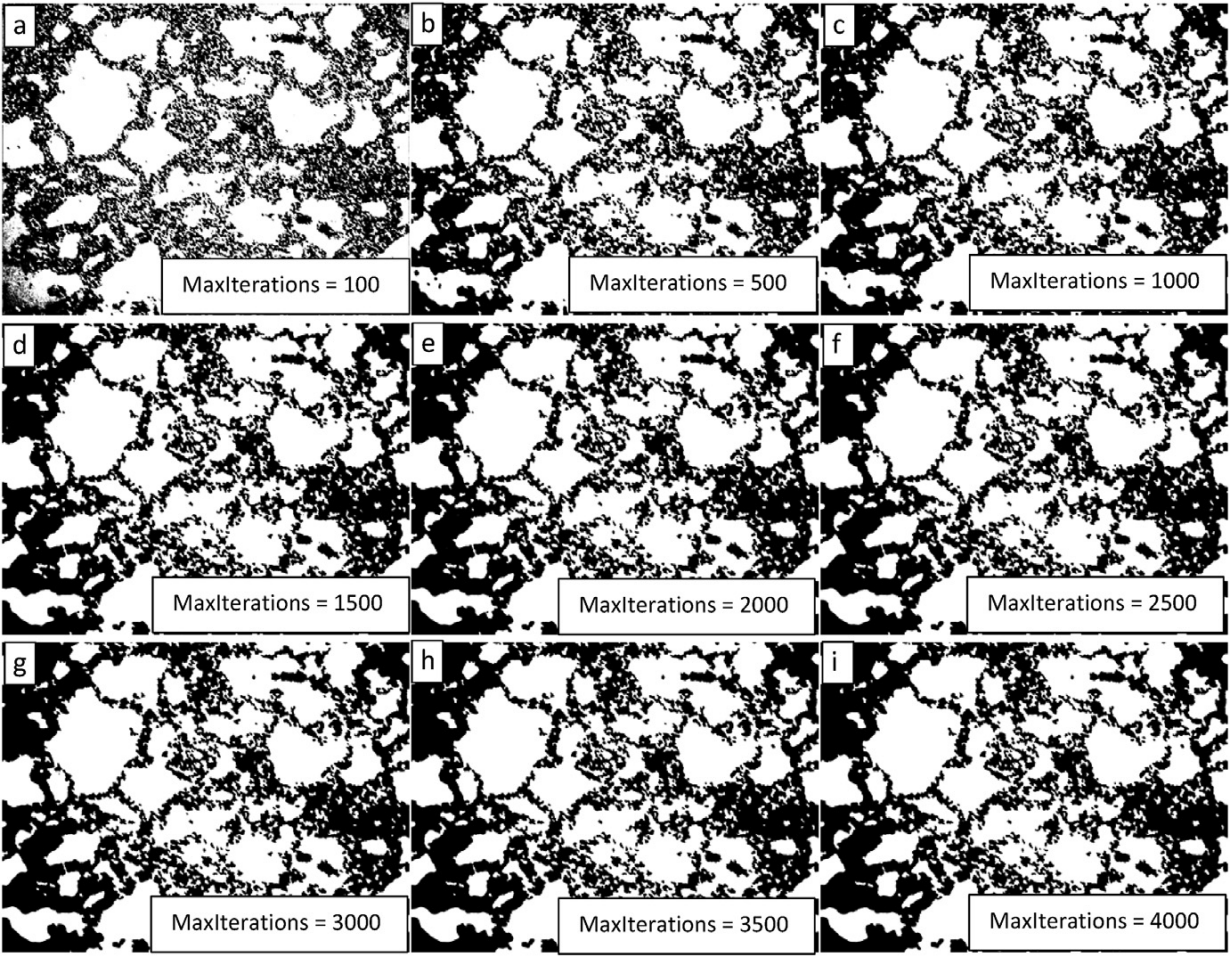
Demonstration of the convergence of the Chan-Vese algorithm on the image shown in Fig. 13 for μ = 0, ν = 0.03, λ_1_ = 1, λ_2_ = 1.0, and MaxIterations varying from 500 to 4000 in steps of 500 (with 100 for comparison) from upper left to lower right (a to i).

Based on the segmentation results of the optical image just discussed, it was determined that cropping off the edges where the undesired intact grain erosion is occuring may result in more efficient image segmentation. The cropped image generated in this process is presented in Fig. 13. Fig. 14 shows the segmented images generated by using the segmentation algorithm discussed here for values of the MaxIterations ranging from 500 to 4000 with 100 for comparison. While some improvement is apparent, the segmentation is not as clean in the center of the image as it is for the image periphery. No combination of adjustments of the level penalty and length penalty resulted in a better segmentation than those presented in Fig. 14.

**Fig. 15:**
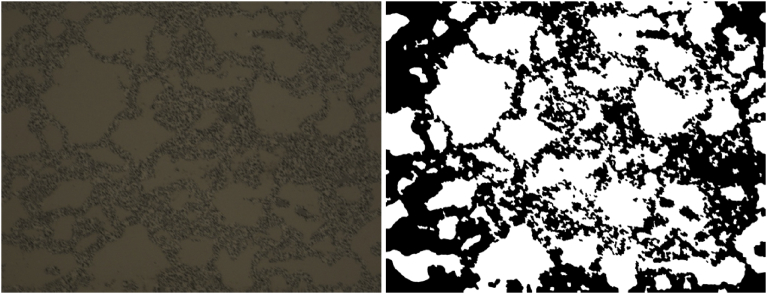
Direct comparison of the original and segmented cropped images obtained through optical microscopy.

A direct comparison of the cropped image in Fig. 13 and the lower-right image from Fig. 14 is shown in Fig. 15. While the segmentation captures many of the features that can be clearly identified as intact grains, the segmentation is not as clean as that obtained for the SEM images.

## 5 Discussion

As was the case when characterizing fission gas bubbles, it is noted that the mechanical preparation relative to preparation with a focused ion beam relieves the necessity for filters to remove curtaining artifacts [5, 6, 7, 8]. The major difference between the characterization of fission gas bubbles and the determination of the degree of grain refinement is the optimal image magnification to be acquired from the microscope leading to ideal results. As higher magnification images yielded interesting results in the characterization of fission gas bubbles, the application of the Chan-Vese contour segmentation algorithm as described in this paper became more and more difficult to use. While this method or other contour segmentation algorithms could no doubt be used successfully, the difficulty presented to the authors counteracted the objective of generating a simple, repeatable baseline method of analysis. Additionally, it is questionable as to whether higher magnification images yield any higher value in the determination of degree of grain refinement than lower magnification images. It is true that higher magnification allows for better determination of the grain boundaries (contours) themselves, but the highly accurate mapping of the contour of a single grain is less useful than a possibly slightly less accurate mapping of the contour of several grains against a large field of refined matrix. In fact, it is possible that a minimum number of existing grains for adequate degree of grain refinement be determined for future use in evaluating the quality of refinement determinations. Even so, the degree of grain refinement determined from the successful segmentation of a 2000x magnification image leads to a local degree of grain refinement that will vary somewhat from the global degree of grain refinement of the entire fuel. Of all the images examined with the Chan-Vese method in this study, it is the lowest magnification images (2000x) that provided the best results as evidenced by ease of use and visual comparison of the original and segmented images.

It is likely that optimal magnification for the use of this method for determination of degree of refinement for a fuel is less than 2000x. When the exercise of image acquisition and segmentation presented in this paper is repeated in the future, high-resolution images with magnifications less than 1000x should be acquired. These images will include more of the fuel meat for analysis and allow for a more representative characterization of the bulk material to be determined from fewer images. Whether the images are acquired through optical microscopy or scanning electron microscopy will be a crucial decision in the execution of the exercise. If the images are acquired through OM, it is extremely important that the illumination of the images be as uniform as that of the SEM images presented in this paper so that the preferential erosion of the periphery of each image is not observed as was the case with the images considered here. If the images are acquired with SEM, the resolution will be limited by the charging and drift associated with these large, highly radioactive samples. The trade-offs must be carefully weighed. Alternatively, as the SEM images acquired at 2000x magnification were found to be adequate for segmentation and analysis by this method, more images of this magnification could be acquired from each sample for increased statistics. However, the decision between acquiring more images or images of lower magnification (and perhaps higher resolution) is influenced by the desire to limit the time that these highly radioactive samples are handled or placed in the microscope.

As the purpose of this work was to define a simple, repeatable baseline method of analysis, the use of the popular Chan-Vese contour segmentation algorithm as pre-built into commercially available software was utilized. However, many other methods may exist and it is possible that other algorithms can be developed to be better suited for use in the determination of degree of grain refinement in irradiated nuclear fuels. The development and application of contour segmentation techniques and algorithms is a very active area. Many of these algorithms are available in commercially available software, leading to the observation that if a higher volume of images like the SEM images presented in this study were available, much progress could be made by individuals investigating the applicability of each newly available algorithm produced in this highly dynamic field.

**Fig. 16:**
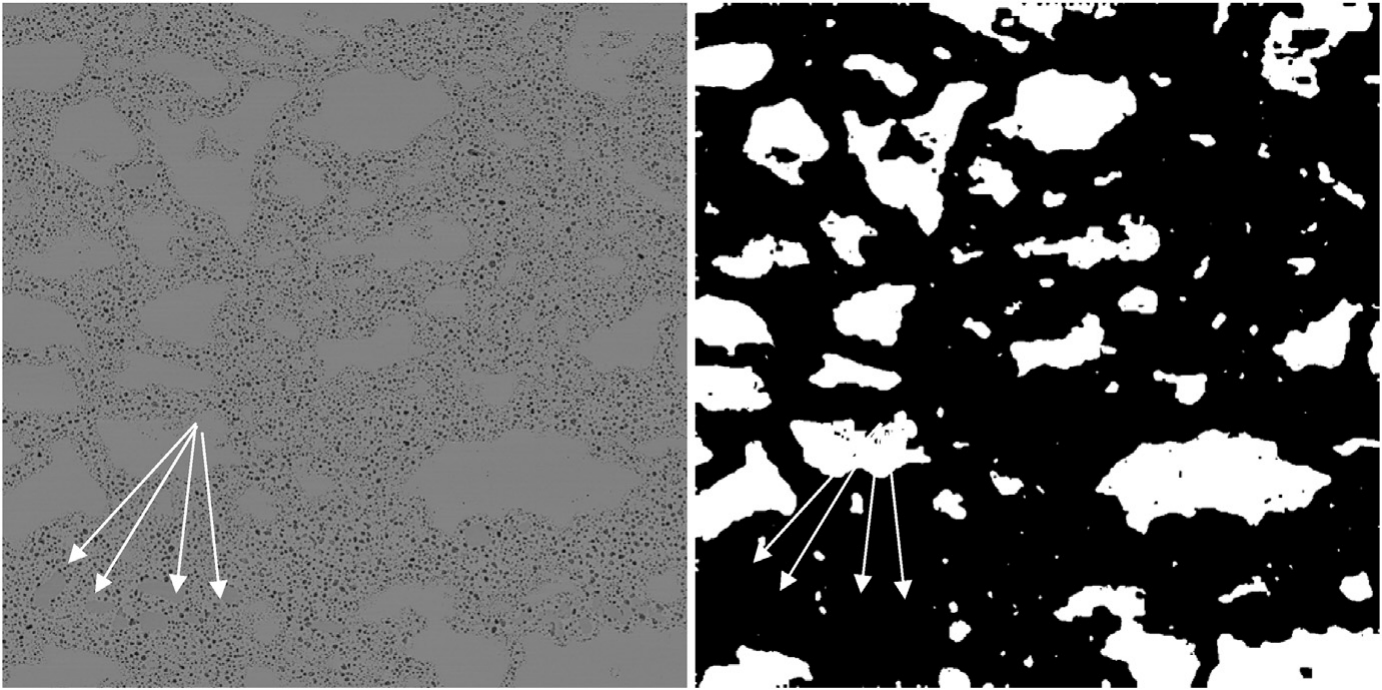
Pre- and post-segmentation images showing the removal of darker impurities.

One interesting property of the process as defined in this paper is that it has the apparent effect of removing material impurities from the image. This is demonstrated in Fig. 16, where structures that appear to be intact grains of a darker shade than other grains are demonstrated by the arrows. These grains are removed by the algorithm. At first, the authors did not notice the difference in shade and thought that the algorithm parameters needed to be changed to allow inclusion of these objects in the final segmented images. However, upon further consideration, it is the opinion of the authors that these objects are material impurities and it is not clear how they should be accounted for during determination of the degree of grain refinement. Confirmation of this hypothesis would need to be verified through EDS.

Beyond consideration of other contour segmentation approaches, there are many additional processing steps that could be considered in an enhancement of the approach presented in this paper. Uses of filters, such as a Gaussian filter, can smooth edges and lead to contours in the image that are easier to segment. On the other hand, filters can also lead to a distortion of the final segmented image and introduce additional complexities in the process.

## 6 Conclusions

This work has introduced the concept of using a well-known contour segmentation algorithm developed by Chan and Vese to differentiate between remaining grains and refined matrix in irradiated U-10Mo fuels. It has been shown that a pre-constructed algorithm within the commercially available Mathematica software can be used to efficiently segment SEM images acquired at 2000x magnification from samples prepared with mechanical potting and polishing techniques. Additionally, it has been recognized that this method starts to become less effective at higher magnifications and is most likely more useful at magnifications less than 1000x. It is possible that optical microscopy images may be optimal for this method, if the image illumination can be kept uniform. The segmented images can subsequently be used to determine the degree of grain refinement of the irradiated fuel rather reliably. It is recognized that other programs that use the Chan-Vese algorithm (such as MATLAB) are also commercially available. Also, many other contour segmentation approaches may be available or be currently under development. Further investigation of this method of image segmentation may generate a more useful set of tools for evaluating irradiated nuclear fuels than are currently available. Fully developing these tools for determining the degree of grain refinement within U-Mo fuels or other fuels that may be considered for future use will prove to be of great use in the characterization of their performance under irradiation and greatly facilitate their qualification for use.

## Declarations

### Author contribution statement

Andrew M. Casella: Conceived and designed the experiments; Performed the experiments; Analyzed and interpreted the data; Contributed reagents, materials, analysis tools or data; Wrote the paper.

Douglas E. Burkes: Conceived and designed the experiments; Contributed reagents, materials, analysis tools or data.

Paul J. MacFarlan: Performed the experiments; Contributed reagents, materials, analysis tools or data.

Edgar C. Buck: Contributed reagents, materials, analysis tools or data.

### Funding statement

This work was supported by the National Nuclear Security Administration's Office of Material Management and Minimization Reactor Conversion Program under the contract DE-AC05-76RL01830.

### Competing interest statement

The authors declare no conflict of interest.

### Additional information

No additional information is available for this paper.
